# Use of Laser Speckle Contrast Analysis during pelvic surgery in a uterine transplantation model

**DOI:** 10.4155/fsoa-2018-0017

**Published:** 2018-08-01

**Authors:** Srdjan Saso, Maria Tziraki, Neil T Clancy, Lipei Song, Timothy Bracewell-Milnes, Benjamin P Jones, Maya Al-Memar, Joseph Yazbek, Meen-Yau Thum, Ahmad Sayasneh, Tom Bourne, James Richard Smith, Daniel S Elson, Sadaf Ghaem-Maghami

**Affiliations:** 1 Division of Surgery & Cancer, Institute of Reproductive & Developmental Biology, Imperial College London, Hammersmith Hospital Campus, Du Cane Road, London, W12 0HS2 UK; 2 Hamlyn Centre for Robotic Surgery, Institute of Global Health Innovation, Imperial College London, London, SW7 2AZ3 UK; 3 Department of Obstetrics & Gynaecology, Chelsea & Westminster Hospital, Imperial College London, London, SW10 9NH4 UK; 4 West London Gynaecological Cancer Centre, Queen Charlotte's Hospital, Hammersmith Hospital Campus, Imperial College London, Du Cane Road, London, W12 0NN6 UK; 5 The Lister Hospital, Chelsea, London, SW1W 8RH7 UK; 6 Guy's & Thomas’ Hospitals, London, SE1 7EH8 9 UK

**Keywords:** graft anastomosis, oxygen saturation, surgical imaging, tissue perfusion, uterine transplantation

## Abstract

**Aim:**

Uterine transplantation (UTx) is proposed for treatment of uterine factor infertility. Our aim was to assess whether Endoscopic Laser Speckle Contrast Analysis (eLASCA) could evaluate pelvic blood flow at anastomotic sites required for sheep and rabbit UTx.

**Results/methodology:**

eLASCA detected blood flow in rabbit UTx #7 and #9. In sheep UTx #2, #3 and #5, the results allowed us to conclude that blood flow was present in the uterine graft following transplantation; and post-UTx, the animal had heart and respiratory rates, and oxygen saturation compatible with a normal hemodynamic status.

**Conclusion:**

These preliminary results establish the potential of Laser Speckle Contrast Analysis as noncontact and real-time tool for observation of spatially-resolved blood flow from which other parameters can be derived.

## Lay abstract

Transplanting wombs into women has been offered as a potential solution to treat women who have had them removed previously or who were born without one. A major problem of the surgery that one may face is how to ensure good blood flow within those vessels supplying the womb. Our aim here therefore was to describe a technology known as Endoscopic Laser Speckle Contrast Analysis, which may offer a solution. The experiments were performed in two types of animals, rabbit and sheep. The use of Endoscopic Laser Speckle Contrast Analysis is the first case in gynecology and has demonstrated promise of possible future human use.

Uterine transplantation (UTx) is a surgical procedure proposed for the treatment of absolute uterine factor infertility, whereby the absence of the uterus renders a woman unconditionally infertile. The feasibility of UTx to offer the potential for pregnancy to women diagnosed with absolute uterine factor infertility has been demonstrated by the first live-birth following UTx [1,2].

A major problem that one may face in a human UTx model is how to ensure an adequate blood flow within those vessels supplying the uterus: immediately postanastomosis, in the early and late postoperative periods, and during pregnancy when those anastomotic sites will be severely tested because of an increase in vessel diameter. Therefore, it is of crucial interest to be able to image the uterine circulatory system that maintains the flow of oxygen and nutrients to the relevant tissue. By investigating tissue circulation, one can make conclusions relevant to tissue oxygenation, and potentially, the level of ischemia and reperfusion injury. The level of oxygenation of an organ is directly proportional to the health of that organ. Currently, there is no standard imaging technique to achieve this.

Biomedical photonics studies the interaction between light and tissue and can provide information on processes occurring at a molecular level, both at microscopic spatial and nanometer spectral resolutions [3,4]. Today, biomedical photonics is a well-established and commonly utilized tool in pulse oximetry, endoscopy, laser therapy, and microscopy. A type of biomedical photonics, known as Endoscopic Laser Speckle Contrast Analysis (eLASCA), could offer a solution to the above problem. It is an imaging system which monitors vessel blood flow by measuring temporal changes in the light reflected from tissue under laser illumination because of the presence of moving ‘scatterers’ (red blood cells) [5]. Currently, eLASCAhas been applied in practice to quantify and assess blood circulation in the skin, retina and cortical tissue [6,7]. However, it has never been utilized in gynecology. The objective of the study was to assess whether eLASCA could be used to evaluate pelvic blood flow at anastomic sites required for a successful UTx in two animal models.

## Materials & methods

Full details of the surgical procedures may be found in separate manuscripts relating to the rabbit [8] and sheep transplants [9]. All animal experiments described in this paper were conducted under UK Home Office licenses (70/7508 and 70/6927). Background information on biomedical photonics and eLASCAis summarized in Appendix A.

The first application of eLASCA was by Forrester *et al*. who used it to image tissue perfusion and blood flow in the medial compartment of the knee of five patients requiring arthroscopic knee surgery. Changes in tissue perfusion were brought about by tourniquet application and intra-articular adrenaline injection, and were then displayed as real-time video perfusion images of tissue blood flow in the knee joint. Only a high-resolution charged couple device (CCD) and a standard arthroscopic sheath with which to cover the rigid Hopkins lens type endoscope were needed for this study [15]. The next study advanced this concept further. Zimnyakov *et al*. applied a fiber-optic bundle and a CCD camera instead of the above rigid endoscope to monitor a speckle-contrast pattern. However, it was a point measurement and could therefore not provide images of the scatter speeds [16].

### The experiment

The eLASCA system is an optical fiber-based probe, which has been described in detail previously [17]. The flexible endoscope consists of two illumination fibers, which carry light from a red and near-infrared laser diode (ML101J27 660 nm and DL8142 830 nm, Thorlabs, NJ, USA), and a central optical fiber bundle (leached fiber image guide; Schott, Mainz, Germany), which collects backscattered light via a miniature objective lens at the tip. The leached fiber image guide then transmits the image of the tissue, and its speckle pattern, to a 12-bit CCD camera (Retiga Exi cooled CCD, Qimaging, British Columbia, Canada) via a magnification system consisting of a microscope objective (×10, Olympus, Tokyo, Japan), and an achromatic lens (*f* = 100 mm, Thorlabs). The field-of-view of the probe was approximately 6 mm^2^. A customized LabVIEW program was used to control alternate switching of the lasers (using a data acquisition card (DAQ); National Instruments, TX, USA) and was synchronized with exposure of the CCD. The slower recording condition was five frames per second (fps) for each wavelength and 10 fps for both. The lasers, magnification system and the CCD camera were set on a portable optical bench which was mounted on a trolley [18]. The stack of raw images was processed, postacquisition, using a custom made software Matlab [19]. Camera exposure times for intra-operative measurements were chosen to optimize detected light intensity while remaining sensitive to physiological flow rates. Exposure time values of 500 μs, 1 ms and 10 ms were used.

**Figure 1.**
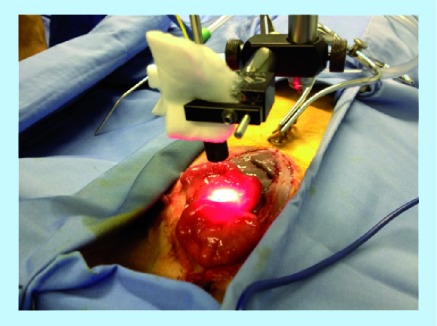
**Measurement site for Endoscopic Laser Speckle Contrast Analysis performed on a sheep uterus.** This photograph was taken shortly after the abdomen was opened and the uterus was exteriorized.

The eLASCA was performed after multi-spectral imaging in both a rabbit and a sheep model [20]. Prior to surgery, heart rate and oxygen saturation (O_2_Sat) were measured using a standard pulse oximeter. For the rabbit transplants, the aim was to carry out eLASCA prior to the recipient hysterectomy only, in order to simply try the technique and apparatus. In the sheep cohort, eLASCA was performed before the graft retrieval and once more, 30 min following the establishment of perfusion (Figure 1).

The experiment for the rabbit animal model consisted of two parts: the detection of the heartbeat (with a single wavelength and fast CCD frame rate); and the detection of the change in blood flow and tissue oxygenation following a vessel occlusion. In the sheep model experiment, no vessels were occluded. In the rabbit experiment, a single laser diode (λ = 660 nm) was turned on. The heartbeat of the rabbit ranges from 130 to 220 beats per minute (bpm). Therefore to capture the structure of the contrast change in one pulse period, the frame rate for the rabbit required the value used in the finger test to be doubled. This was not the case with the sheep experiments, where the heart rate is comparable to the human. This, in turn, meant a decrease in the exposure time leading to a fall in the signal intensity and spatial resolution. In order to limit the effects of the latter two, the exposure time was set to 0.5 ms, thus reaching a compromise between the frame rate and the intensity. A total of 30 frames were recorded at a time, the process lasting 1.4 s. In the latter experiment, both laser diodes were used. Data were acquired initially for 25 s, with normal circulation throughout; subsequently the artery was occluded for 25 s while the CCD continued to record images. Finally, the artery was released and the speckle images were recorded for another 15 s. The exposure time was 20 ms and the average frame rate was 1.6 fps for each wavelength. The acquisition time of every frame was also recorded into an Excel document. A customized Labview program controlled and synchronized the laser switch, the filter wheel and the camera acquisition.

Oxy-hemoglobin and deoxy-hemoglobin have different absorption coefficients at most wavelengths and can be therefore used to detect the hemodynamics of blood circulation. Oxygenized blood is supplied continuously to the tissue whereas deoxygenized blood flows back to the heart. When the blood flow is stopped in the vessels, the concentration of the oxy- and deoxy-hemoglobin varies which results in a change in the light intensity absorbed and reflected back to the blood. Therefore the blood supply can be monitored by the change of the intensity of the reflected light. The modified Beer–Lambert law is used to calculate the change of the oxygenation [6,20]. In this experiment two wavelengths, 660 and 830 nm, were used. The oxygenation image is ratiometrically calculated by the images at wavelengths of 660 and 830 nm based on the difference between absorption coefficients of the tissue chromophores.

## Results

### Rabbit model (UTx #7 & #9)

#### Temporal analysis & occlusion study

**Figure 2.**
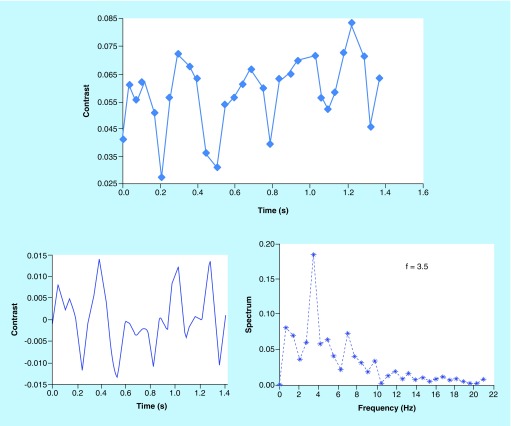
**Graph showing contrast portrayed as a function of frames (data acquired at 660 nm). The contrast and the spectrum of the contrast.** **(A)** Graph showing raw contrast as a function of time. **(B)** Graph showing contrast after detrending and interpolation. **(C)** Graph showing frequency spectrum of the interpolated contrast.

The average contrast from the area of interest was calculated for each frame and plotted as a function of time (Figure 2A). This signal was then detrended to remove the direct current component and interpolated with a double sampling frequency (Figure 2B). The contrast displayed a periodic change. Then a fast Fourier transform was applied to the resulting contrast used to calculate the frequency at which the contrast varied. The frequency spectrum of the contrast is shown in Figure 2C. The peak frequency was around 3.5 Hz, corresponding to 210 bpm, which is slightly higher than the 160–180 bpm measured using an oximeter at the commencement of the trial.

**Figure 3.**
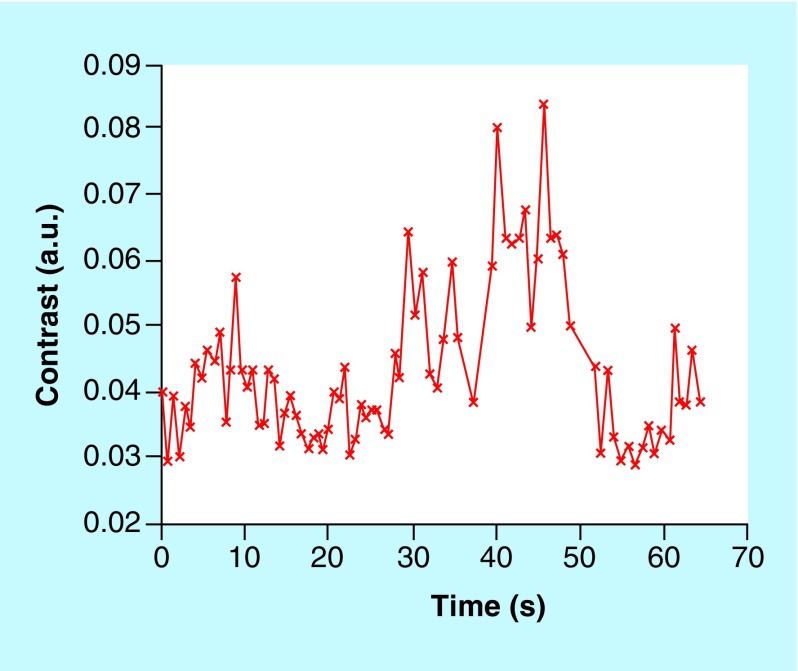
**Graph showing contrast portrayed as a function of frames (data acquired at 660 nm).**

**Figure 4.**
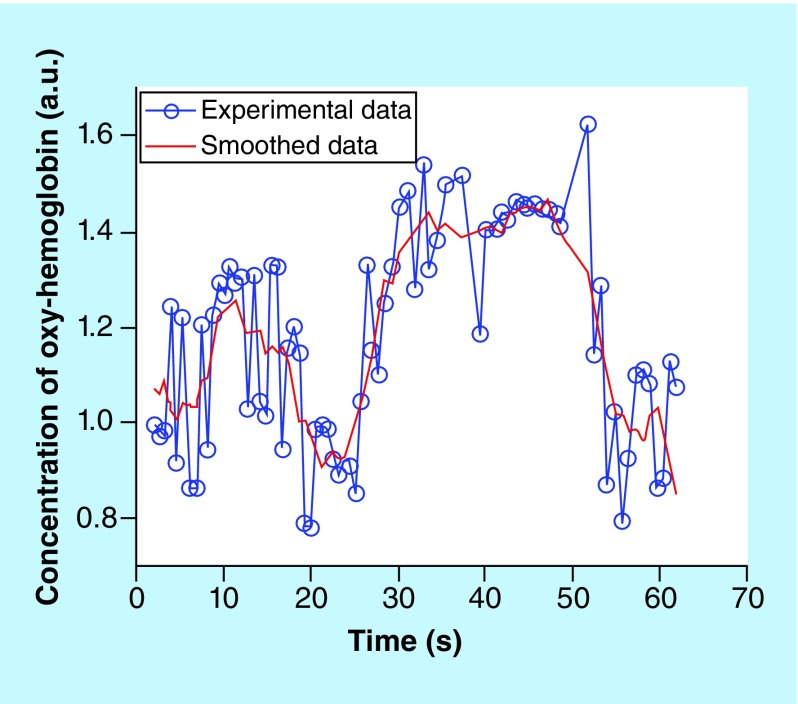
**Graph showing the variation of oxy-hemoglobin concentration with time.**

Figure 3 demonstrates the contrast change using both wavelengths, following the occlusion of the aorta. The two figures depict a matching increase in contrast after the 25th second when the occlusion was applied, thus confirming that the blood flow was blocked, and a drop in contrast at the 50th second when the blood flow had been recommenced. The spikes show a periodic change of 2 s, most likely brought about by the breath cycle as the respiration rate of the rabbit is 30–60 breaths per minute. The variation of oxy-hemoglobin concentration with time is shown in Figure 4.

**Figure 5.**
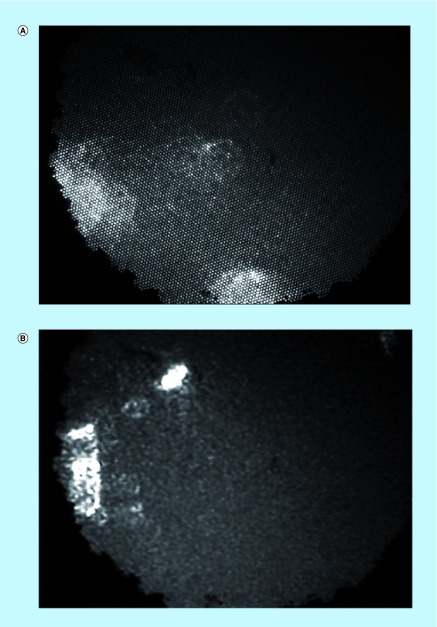
**Fibre bundle pattern applied and removed.** **(A)** Focused and imaged fiber bundle pattern in the donor. **(B)** Image of interest when the low pass Butterworth filter was applied. It removed the fiber bundle pattern thus ensuring that the contrast is mainly from the fiber pattern and not from the sample.

Similar results were found for rabbit UTx #9. eLASCA was shown to be able to localize a group of high flow scatterers which are red blood cells containing both oxygenated and deoxygenated hemoglobin. Figure 5A shows a focused and imaged fiber bundle pattern in the donor (exposure time 10 ms; frame rate: about 21 fps; frame: 0–199 frame) and Figure 5B an image of interest when the low pass butterworth filter was applied. It removed the fiber bundle pattern, thus ensuring that the contrast is mainly from the fiber pattern and not from the sample.

### Sheep model

We present the results of the three autotransplants that survived: #2, #3 and #5.

#### Sheep experiment - UTx #2

##### Preretrieval

**Figure 6.**
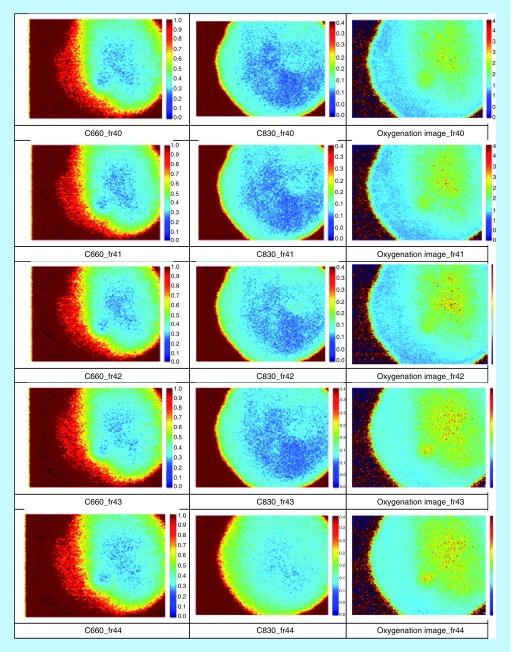
**Image acquisition preretrieval.**

Figure 6 shows image acquisition preretrieval where the exposure time of the camera was set to 1 ms. The Speckle Contrast images at the corresponding wavelengths (C660 and C830) demonstrate blood circulation, which is depicted by the blue areas. The lower the contrast (darker blue area) the faster is the blood flow. The oxygenation image was calculated by taking the ratio of the same frame at the two different wavelengths. At the resulting image (third column) areas of relatively high blood oxygenation are indicated by the color red. These results are qualitative and show that the speckle contrast images are a good indicator of blood flow.

**Figure 7.**
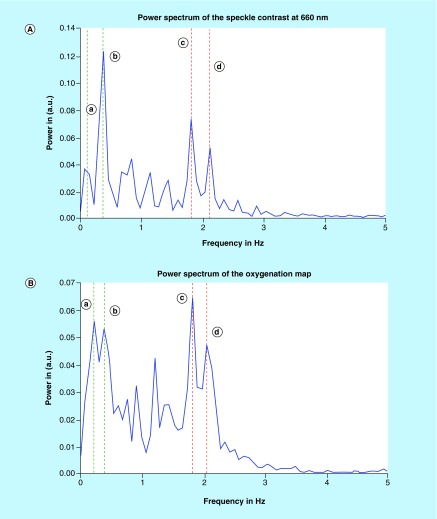
**(A) Intensity distribution of the various frequencies of speckle contrast at 660 nm.** **(a)** 0.13 Hz attributed to a breathing rate of 7.8 breaths per min **(b)** 0.38 Hz attributed to a breathing rate of 22.8 breaths per min or to any vibration of the system **(c)** 1.83 Hz attributed to a cardiac rate of 109 bpm **(d)** 2.13 Hz attributed to a cardiac rate of 127 bpm. **(B)** Intensity distribution of the various frequencies of the oxygenation map. **(a)** 0.23 Hz attributed to a breathing rate of 13.8 breaths per min. **(b)** 0.38 Hz attributed to a breathing rate of 22.8 breaths per min or to any vibration of the system. **(c)** 1.815 Hz attributed to a cardiac rate of 109 bpm **(d)** 2.08 Hz attributed to a cardiac rate of 123.6 bpm.

**Figure 8.**
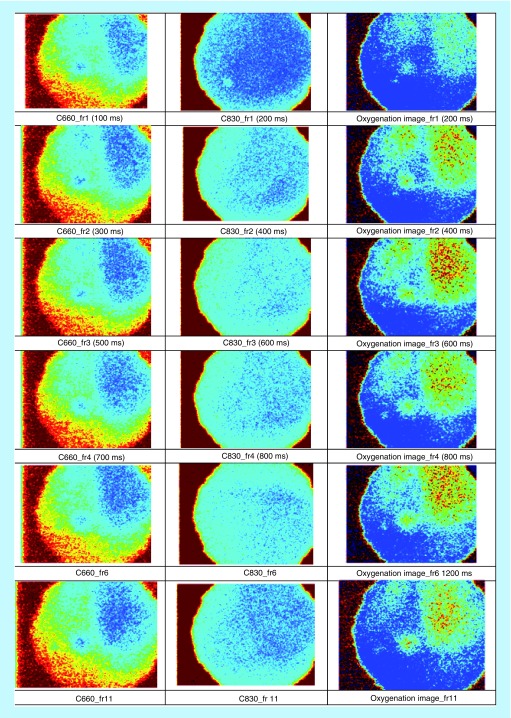
**Image acquisition post-transplantation (UTx #2).**

**Figure 9.**
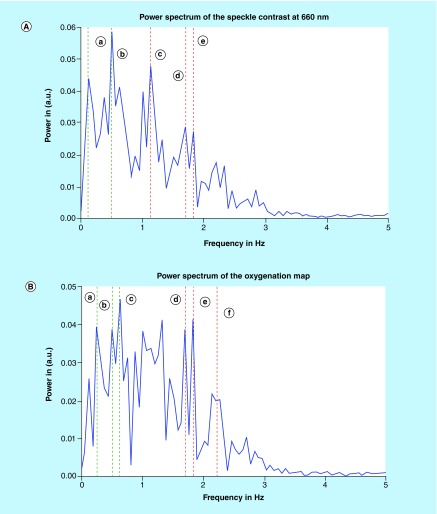
**(A) Intensity distribution of the various frequencies of speckle contrast at 660 nm.** **(a)** 0.13 Hz attributed to a breathing rate of 7.8 breaths per min **(b)** 0.50 Hz attributed to a breathing rate of 22.8 breaths per min or to any vibration of the system **(c)** 1.15 Hz attributed to a cardiac rate of 69 bpm **(d)** 1.70 Hz attributed to a cardiac rate of 102 bpm **(e)** 1.82 Hz attributed to a cardiac rate of 109 bpm. **(B)** Intensity distribution of the various frequencies of speckle contrast of the oxygenation map. **(a)** 0.27 Hz attributed to a breathing rate of 16.2 breaths per min **(b)** 0.51 Hz attributed to a breathing rate of 30.6 breaths per min or to any vibration of the system **(c)** 0.62 Hz attributed to a cardiac rate of 37.2 bpm **(d)** 1.72 Hz attributed to a cardiac rate of 103.2 bpm **(e)** 1.81 Hz attributed to a cardiac rate of 108.6 bpm **(f)** 2.22 Hz attributed to a cardiac rate of 133.2 bpm.

Detection of heart and respiratory rates is indicated in the power/frequency plot shown in Figure 7. The peaks visible at 0.38 and 1.83 Hz correspond to 22.8 and 109 bpm and are consistent with motion due to the cardiac cycle and respiration, respectively. Image acquisition post-transplantation is depicted in Figure 8. Again the calculation of heart and respiratory rates post-transplantation is noted in Figure 9A and Figure 9B.

**Figure 10.**
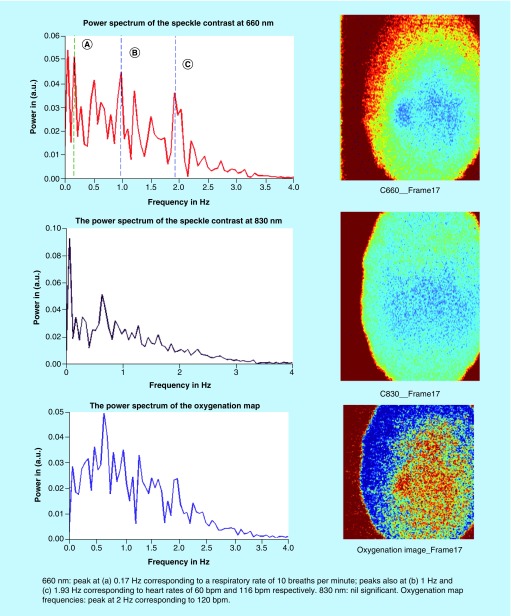
**Preretrieval speckle contrast results (UTx #3).**

**Figure 11.**
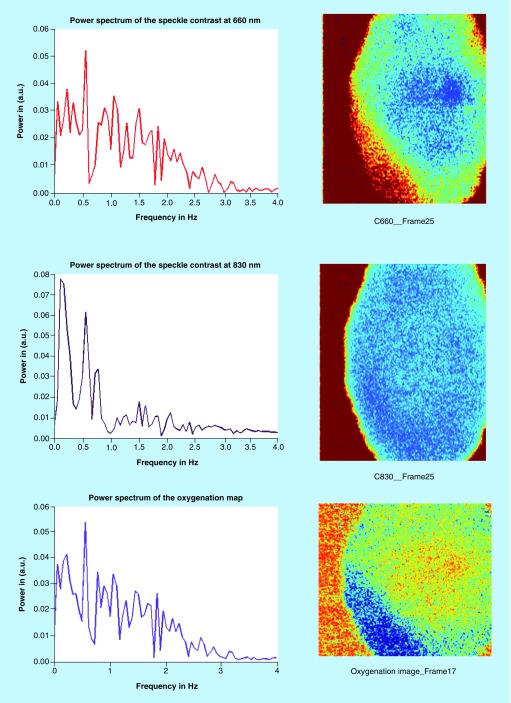
**Postretrieval speckle contrast results (UTx #3).**

**Figure 12.**
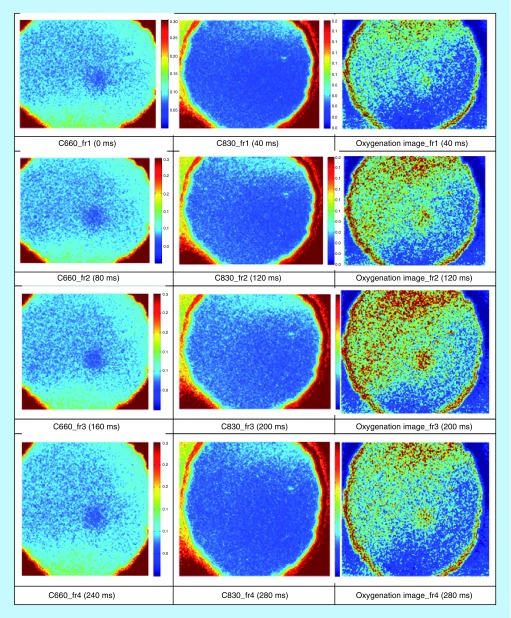
**Preretrieval speckle contrast results (UTx #4).**

**Figure 13.**
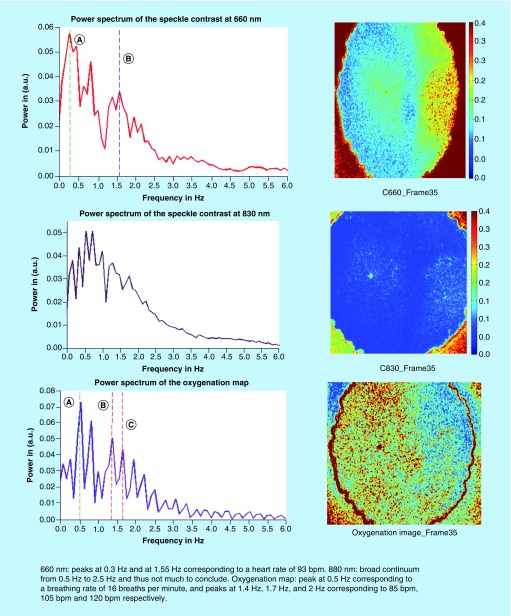
**Preretrieval speckle contrast results (UTx #5).** bpm: Beats per minute.****

**Figure 14.**
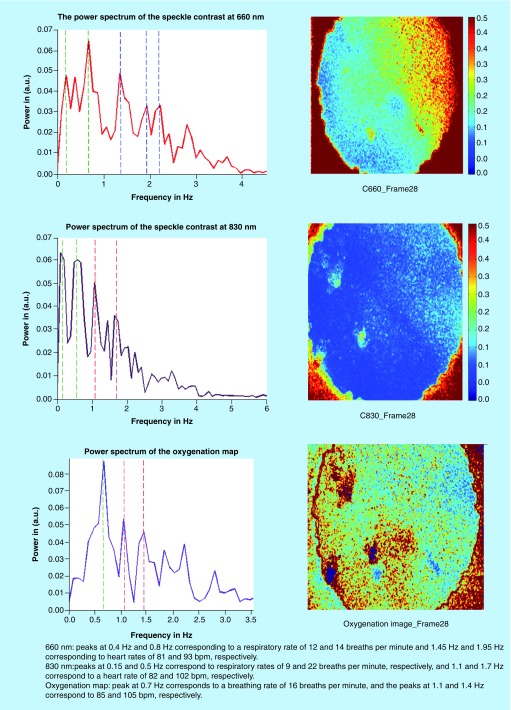
**Postretrieval speckle contrast results (UTx #5).**

For the next three transplants, we will only summarize our results in a single figure per transplant. For more information, similar to what has been described above for UTx #2, please refer to Appendix B. Figures 10 and 11 refer to UTx #3, Figure 12 to UTx #4 and Figures 13 and 14 to UTx #5. In Sheep UTx #4, only the preretrieval data was recorded as the ewe arrested before retrieval commenced. CCD exposure time was set to 20 ms, resulting in an overall acquisition speed of 10 fps.

## Discussion

The ultimate aim of UTx is to enable a feasible allogeneic transplant with respect to anatomy and vascular viability with the subsequent goal to allow embryo transfer and pregnancy at a later date. Our aim here was to see whether a novel technique, eLASCA, could primarily assess uterine blood flow and circulatory function and subsequently, use that data to provide information on other crucial physiological variables: respiratory and heart rates and O_2_Sat. To our knowledge, this is the first time that eLASCA has been attempted in the field of gynecology.

### Main findings

From the experiments, one can extrapolate certain clear advantages when applying LASCA: minimal trauma to the patient, generation of immediate data and highly detailed resolution. eLASCA was applied in UTx #7 and #9 (our rabbit cohort totalled nine). It was not possible to perform it in UTx #8 as a result of recipient demise. With respect to the sheep studies, the aim was to perform eLASCA in all five autotransplants. However, it was only attempted preretrieval and post-UTx in UTx #2, #3 and #5, and preretrieval only in UTx #4 because the first autotransplant was abandoned and the fourth ewe demised very early during the surgery.

The figures demonstrate contrast and intensity plotted against time, with the power spectrum plotted against frequency. For UTx #9, the range of results increased as two exposure times were used for the rabbit does: 500 μs and 10 ms, with frequency set at 0.5 Hz. In Figure 4, which refers to UTx #7, the oxy-hemoglobin concentration, as well as the total hemoglobin, increased following active cessation of arterial blood flow. In theory, the opposite is supposed to occur. The main reason for this may be that during the occlusion of the abdominal aorta, a significant section of the inferior vena cava was also occluded as the two major vessels are found adjacent and parallel to each other. This would then result in a greater proportion of venous blood flow rather than the arterial being blocked off during the occlusion because the uterine artery and the uterine vein are parallel with each other and the vein is close to the surface. The experiment had to be curtailed because the rabbit became hemodynamically unstable. Therefore, the rabbit's abdomen had to be closed promptly, allowing for no time to repeat the results. In UTx #9, when exposure was 500 μs, the intensity of the image, as well as the contrast, fell with identical trends. However here the frequency of the contrast peaked at 2.5 Hz, which allows us to calculate the heart rate: (2.5 × 60) 150 bpm. When exposure was 10 ms, no obvious frequency peak was seen in the contrast frequency. This is most likely because of too long exposure time resulting in an inability to catch the contrast changes induced by the heartbeat. An alternative explanation could be that at longer exposure times, lower frequency motion became more dominant while the higher frequencies, which represent motions of higher speed were washed out.

In order to detect increased heart rates using the contrast versus time data, for example 200 bpm, the frame rate needed to be set higher than 27 fps (however the measured frame rate was 22 fps). Therefore the normal triphasic change associated with a single heartbeat could only be captured when the frame rate was adjusted accordingly. Also, the light-tissue interaction is complex. In a single cardiac period, blood volume, blood flow speed and O_2_Sat can all alter, and additionally, the vessel walls move [20]. All of these factors may potentially affect the contrast value. Therefore the heart beat induces the frequency of the contrast change but other cardiac-related periodic changes in the blood circulation may also contribute to this. Future experiments can be improved by adding a well-established system to monitor the change of O_2_Sat simultaneously and thus compare the two sets of results, one obtained from an established system and the other from a trialled procedure.

The sheep model differed to the rabbit model. The aim in the rabbit model was to see whether eLASCAcould pick up blood flow in the pelvis of a small animal model. In the sheep, the experiment was set up as would be in a human model in order to test whether eLASCAmay have some use in human UTx. Therefore measurements were taken prior to retrieval and post-transplantation, and occlusion was unnecessary. The oxygenation saturation images are ratio-metrically calculated by the images at 660 and 830 nm based on the difference of the absorption coefficients of the oxy and de-oxy hemoglobins. In UTx #2, #3 and #5, the results obtained allow us to conclude that (a) the blood flow was present in the uterine graft following transplantation; (b) at post-UTx, the animal had heart and respiratory rates, and O_2_Sat compatible with a normal hemodynamic status, and (c) at pre-retrieval and post-UTx O_2_Sat of the tissue was comparable. This last point confirms the findings from the multi-spectral imaging.

### Limitations

The main drawback of the study is that we used an animal model and have yet to test the equipment on a human subject. In addition, the number of animals was small (less than ten with both sheep and rabbits). We have therefore not applied a quantitative statistical analysis to assess blood flow or perfusion level changes during the different surgical stages. Hence, the eLASCA system currently remains a prototype for further experimental investigation. The data acquired was limited and difficult to use in order to make any definitive conclusions.

Other challenges are mostly related to the data acquisition methodology. The movements of the target sample *in vivo*, brought on by pulsation, respiration or uterine contraction, lead to faulty contrast images with a low ‘signal-to-noise ratio’. A faster acquisition system is required for more precise diagnostic information. Further improvement of the hardware and the synchronization of the various electronic devices may be the answer.

### Interpretation

LASCA and intra-operative optical measurements in general, offer quite obvious advantages to current imaging techniques. First, the technique is atraumatic to the patient, whereas the ionizing radiation used in medical imaging is harmful and the biopsy involves tissue cutting and therefore damage. Second, optical imaging techniques can generate real-time data during an operation which therefore speeds up diagnosis and subsequent management. With medical imaging and biopsy, one is forced to wait for a period of time until the data is processed. Third, the resolution of optical imaging techniques is highly detailed, with the ability to characterize tissue in the micrometric range and thus, probe biochemical alterations that prestage pathology. This is definitely not the case in ionized medical imaging and biopsy, where a clear demarcation line between diseased and healthy tissue is often unclear [20]. Finally, this data gives us information related to presence of adequate blood flow to a particular tissue, and from that information, heart and respiratory rates and O_2_Sat are calculated. The latter parameter is given in a numerical and a pictorial form.

## Future perspective

These preliminary results demonstrate the potential of eLASCAto surgery and importantly UTx. The system is, in principle, a noncontact and real-time tool for the observation of spatially-resolved blood flow from which other parameters can be derived: heart rate, respiratory rate and O_2_Sat (level and map). Therefore its application is both qualitative and quantitative. Its strengths were revealed when applied to the sheep model. However, there are still many engineering challenges that need to be resolved before it can be reliably introduced into clinical practice.

**Summary points**

- Uterine transplantation has been proposed as a treatment for permanent absolute uterine factor infertility.
- A major problem that one may face in a human uterine transplantation model is how to ensure an adequate blood flow within those vessels supplying the uterus: immediately postanastomosis, in the early and late postoperative periods, and during pregnancy when those anastomotic sites will be severely tested because of an increase in vessel diameter.
- The objective of the study was to assess whether Endoscopic Laser Speckle Contrast Analysis could be used to evaluate pelvic blood flow at anastomic sites required for a successful uterine transplantation in two animal models.
- The use of Laser Speckle Contrast Analysis is the first such case in gynecology and has demonstrated promise of possible future use in humans.
- This data gives us information related to presence of adequate blood flow to a particular tissue, and from that information, heart and respiratory rates and oxygen saturation are calculated.
