# Supplementary material for: Use of Laser Speckle Contrast Analysis during pelvic surgery in a uterine transplantation model
Source: Future Sci OA. 2018 Aug 1;4(7):FSO324. doi: 10.4155/fsoa-2018-0017 (PMC6088268; doi:10.4155/fsoa-2018-0017)
Supplement: Supplementary file 1 [file fsoa-04-324-s1.docx]

**Appendix A**

When light interacts with tissue, it may be absorbed, scattered, transmitted, diffusely reflected or induce fluorescence. Biophotonic imaging techniques seek to quantify the interaction of light with tissue, using appropriate illumination and detection hardware, in order to extract clinically useful information. When striking the surface of an organ some light will be reflected, not penetrating the tissue and therefore not contain significant diagnostic information. This is referred to as a specular reflection. However, the rest of the light may enter the tissue where it will interact with cells, structural fibres and various molecules. In doing so, it will be scattered randomly and absorbed by these constituents, to a degree determined by the wavelength of the light. The fraction of the photons which emerge from the tissue after multiple scattering events are called diffuse reflectance as the scattering induces the spreading and the loss of directionality of the incident beam. Therefore, analysis of the spectral and temporal properties of this reflected light enables assessment of the tissue’s properties.

Biomedical photonic techniques can have a number of advantages. First, diagnostic light levels are atraumatic to the patient, whereas ionising radiation used in conventional medical imaging might be harmfull, and biopsy involves tissue cutting and therefore damage. Second, in contrast to the delay associated with conventional biopsy and histopathology, biomedical photonics techniques have the potential to generate data intraoperatively, speeding up diagnosis and subsequent management. Third, optical imaging techniques could have high spatial resolution (<1 mm) as well as being sensitive to functional and structural aspects of the tissue, which can in turn aid diagnosis. This is not the case in medical imaging and biopsy, where a clear demarcation line between diseased and healthy tissue is often unclear.

*eLASCA*

Parameters of interest with regards to blood flow specifically are blood pressure, blood volume and blood speed. Blood pressure can be easily assessed using a sphygmomanometer; however, the measurement is systemic and not a reading of the blood flow speed in a particular area. Blood volume can be measured using electrical impedance plethysmography and chamber plethysmography. The former has poor accuracy as the method is based on three assumptions which are often not met: the vessels expand uniformly, the resistivity of the blood remains constant, and the direction of the current is parallel to the flow. The latter depends on the blockage of the venous flow and measurement of the volume, and it is therefore only applicable to the limbs, and not the internal organs. Finally, blood flow speed can be assessed by a number of different methods: electromagnetic flowmeters, ultrasound-Doppler flowmeters, and optical methods such as photoplethysmography, laser Doppler flowmetry and imaging, photoacoustic methods and optical coherence and Doppler tomography. However, none of the methods above is able to assess in combination blood flow velocity, blood function, vascular anatomy and tissue perfusion. Thus, the measurement of three variables in particular: blood flow speed, haemoglobin concentration ([Hb]) and oxygen saturation (O_2_Sat).

A laser speckle pattern is produced when a rough area, surface or pattern is illuminated by coherent laser light and is imaged onto a camera (**Figure 1**). Randomly distributed bright and dark granular patterns are created on the observation plane, as a result of the the interference of backscattered light. The resulting grainy image is called a speckle pattern. When imaging an object where the light scatterers are in motion, the speckle pattern that is generated at each pixel varies with time and space. These variations contain flow information about the motion of the scattering particles which can be calculated by measuring either the temporal intensity fluctuation of a speckle (laser-Doppler flowmetry) or the spatial intensity fluctuation (LASCA). If a camera is used to capture an image of the pattern, then individual speckles will appear blurred if their motion is significant over the exposure time. The contrast of the speckle against the background then decreases with increases in the speed of the scatterers. Therefore the speed can be calculated by examining the change of the contrast value which is the basic principle of LASCA [10].

Speckle contrast is generally described as the ratio of the standard deviation over the mean intensity of a small area in the speckle image:

$$C=\frac{\sigma}{\left\langle I \right\rangle}$$

where *C* is the contrast, $\sigma$ is the standard deviation of the signal and $<I>$ is the mean intensity value. In experiments, the contrast is calculated based on the values recorded by the CCD pixels. Usually, in an ROI defined by ‘*n*’ pixels, the mean intensity and the standard deviation are calculated and the speckle contrast number is allocated for this area.

When an object moves, the speckle pattern it produces changes. For small movements of a solid object the speckle pattern moves as a whole, i.e., the speckle pattern remains correlated. For faster motions, the speckles ‘decorrelates’ and the speckle pattern changes completely. Decorrelation also occurs when the light is scattered from a large number of individual moving scatterers, such as particles in a fluid. An individual speckle appears as if it is ‘twinkling’ like a star. This phenomenon is known as a ‘time-varying’ speckle. *Stern at al* first recognised that the biggest potential for the application of speckle pattern fluctuations could be in assessing the flow of red blood cells [11].

Speckle images of tissues *in vivo* can be acquired by fast cameras as series of snapshot images with exposure times as short as 1/1000 sec (1 ms) over a total duration of several seconds or minutes. The contrast calculation is based on a single frame image processing, which is further applied to the whole stack of images with image processing algorithms. For a single digital frame the computer program selects a window of an area of 5×5 pixels or 7×7 pixels known as ‘kernel’ and calculates *C.* The “kernel’ then moves by a pixel step across the digital image and the process is repeated. The result is the speckle contrast image. Here we used 5 x 5 pixels.

Theoretically, the speckle contrast has values between 0 and 1. A completely stable object maximizes the speckle contrast to 1 indicating that there is no blurring and therefore no motion, while moving particles with certain speed results in blurred speckle images with lower contrast values. The faster the motion, which in the case of red blood cells is the blood flow, the ‘darker’ the speckle contrast. Pseudo-colour may be added to the contrast map according to the contrast value so that the different speeds can be distinguished by different colours.

The application of LASCA is limited however because laser light can be absorbed and then scattered whilst passing through tissue. Therefore, the maximum penetration depth is several millimetres. A solution is the combination of LASCA with endoscopy (eLASCA) which allows imaging of internal organs for clinical diagnosis and then surgery if deemed necessary, assessment of the recovery from disease or injury, as well as the acquisition of real-time information of the *in vivo* organ blood supply. Variations in cardiac and respiratory rates can be extrapolated from analysis of the signal over time using Fourier analysis to determine the frequency components of the signal. Furthermore, if more than one wavelength is used, it is possible to employ spectral analysis techniques to determine blood oxygenation status based on known differing absorption properties of oxygenated and deoxygenated haemoglobin [12,13]. Such information will improve upon current imaging and non-imaging techniques for post-operative tissue monitoring, and will, therefore, analyse uterine ‘well-being’ both in its non-pregnant and pregnant state, assess recovery and understand haemodynamics and oxygenation levels. It is therefore potentially a system which may be capable of detecting tissue perfusion, oxygenation level, pulsation and respiration at the same time [5,14].

Figure 1:

| I |  |
| --- | --- |
| **(a)** | **(b)** |
