# Supplementary material for: Use of Laser Speckle Contrast Analysis during pelvic surgery in a uterine transplantation model
Source: Future Sci OA. 2018 Aug 1;4(7):FSO324. doi: 10.4155/fsoa-2018-0017 (PMC6088268; doi:10.4155/fsoa-2018-0017)

**Sheep UTx #3**

The following set of data is recorded with illumination set at 660nm, exposure time at 1ms and delay at 40ms. This corresponds to acquisition frame rate of 25fps. The faster recording allows better sampling, therefore more precise frequencies measurements.

**Figure A** Power spectrum of the frequencies of the speckle (pre-retrieval, ‘donor’). Peaks can be seen at: (a) 0.51Hz corresponding to a respiratory rate of 16 breaths per minute; (b) 1.24Hz corresponding to a heart rate of 75bpm; and (c) 2.12Hz corresponding to a heart rate of 135bpm.


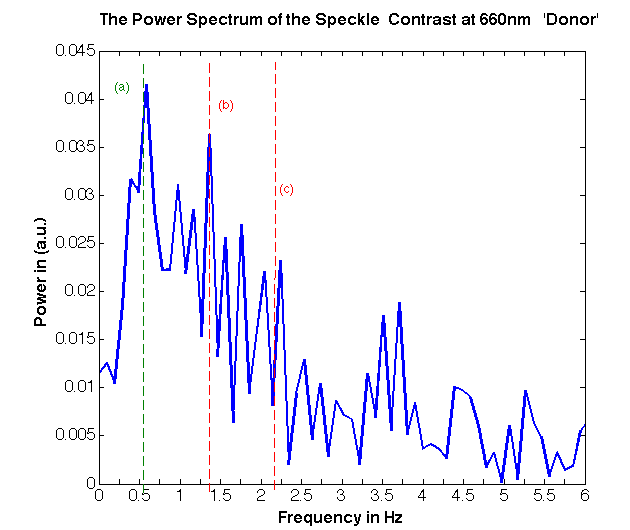


**Figure B** Power spectrum of the frequencies of the speckle (post-transplant, ‘recipient’). Peaks can be seen at: (a) 0.32Hz corresponding to a respiratory rate of 19 breaths per minute; (b) 1.16Hz corresponding to a heart rate of 70bpm; and (c) 2.18Hz corresponding to a heart rate of 131bpm.


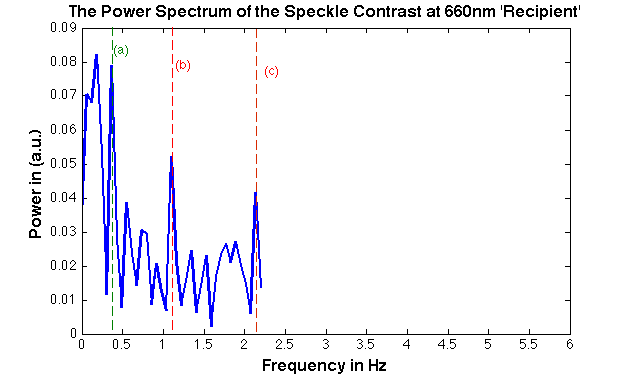


**Sheep UTx #4**

**Figure C** Intensity distribution of the frequencies of the speckle as calculated when applying all data. Peaks can be seen at: (a) 0.35Hz corresponding to a heart rate of 21bpm; (b) 0.675Hz corresponding to a heart rate of 40bpm, and (c) 1.75Hz corresponding to a heart rate of 105bpm.


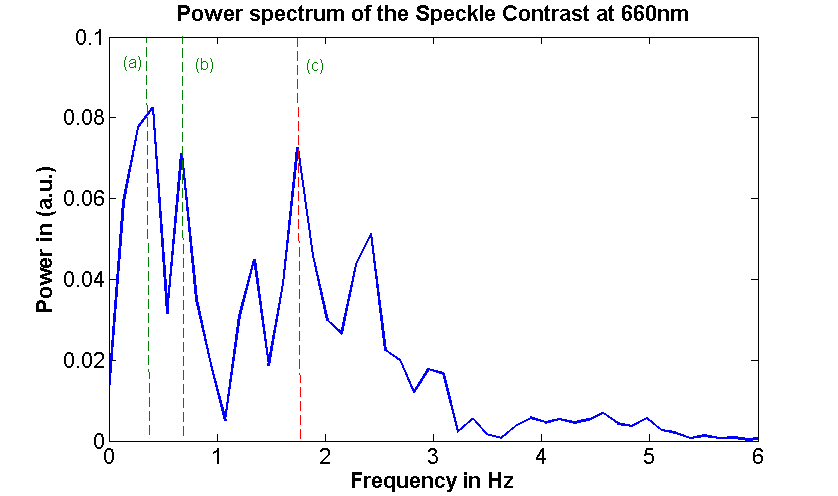


**Figure D** Peaks can be seen at: (a) 0.15Hz corresponding to a respiratory rate of 9 breaths per minute; (b) 0.8Hz corresponding to a heart rate of 48bpm and (c) 1.57Hz corresponding to a heart rate of 94bpm.


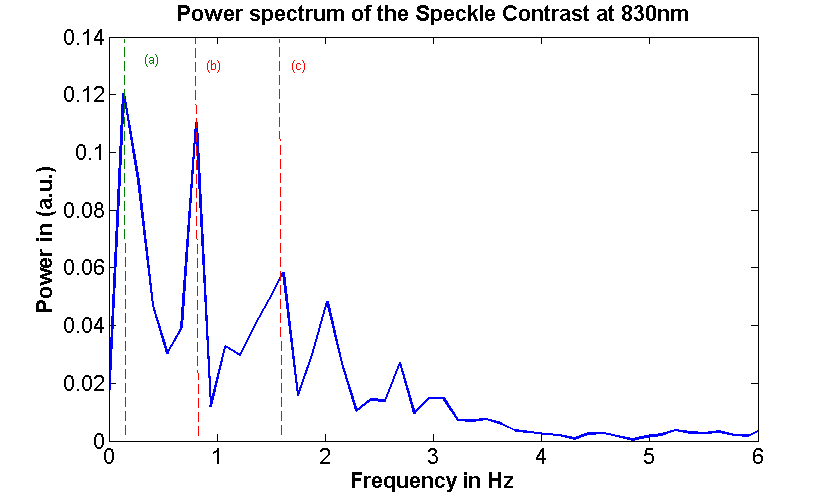


**Figure E** Intensity distribution of the speckle frequencies as derived from the oxygenation map images. Peaks can be seen at: (a) 0.13Hz corresponding to a respiratory rate of 7.8 breaths per minute; (b) 0.4Hz corresponding to a heart rate of 24bpm; and (c) 0.8Hz corresponding to a heart rate of 48bpm.


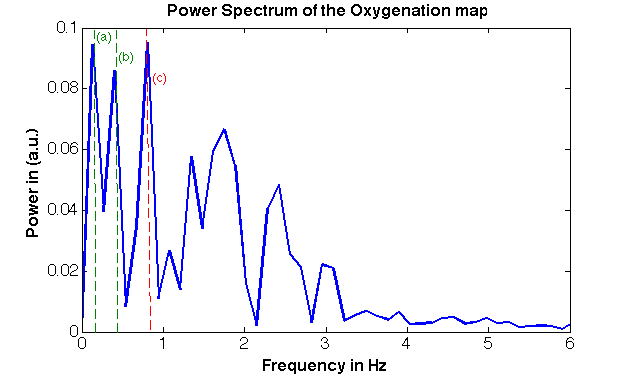

Supplement: Supplementary file 2 [file fsoa-04-324-s2.docx]
